# Supplementary material for: Multi-stimuli-responsive programmable biomimetic actuator
Source: Nat Commun. 2019 Sep 9;10:4087. doi: 10.1038/s41467-019-12044-5 (PMC6733902; doi:10.1038/s41467-019-12044-5)
Supplement: Supplementary file 1 — Supplementary Information [file 41467_2019_12044_MOESM1_ESM.pdf]

Supplementary Information

Multi-stimuli-responsive Programmable Biomimetic Actuator

Y. Dong et al.

Supplementary Figures

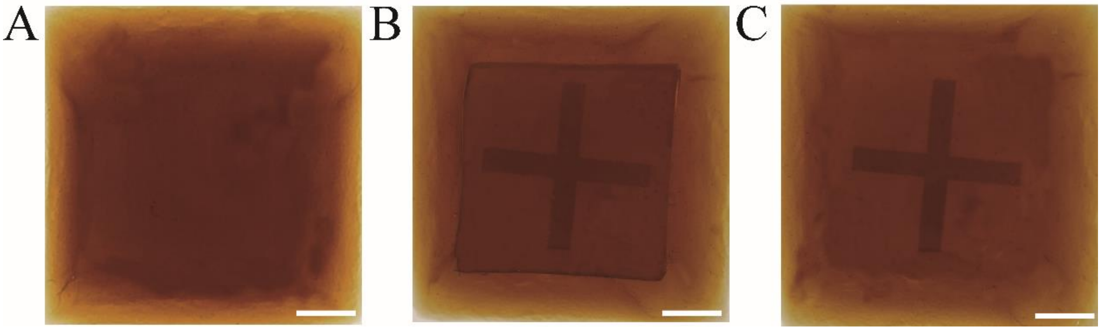

Supplementary Figure 1. The formation of aqueous film of $FeCl_3$ with specific patterns on GO film. The photographs of (A) GO film, (B) $FeCl_3$-loaded agarose hydrogel stamp on GO film and (C) water film with cross pattern on GO film (Scale bar: 1 cm).

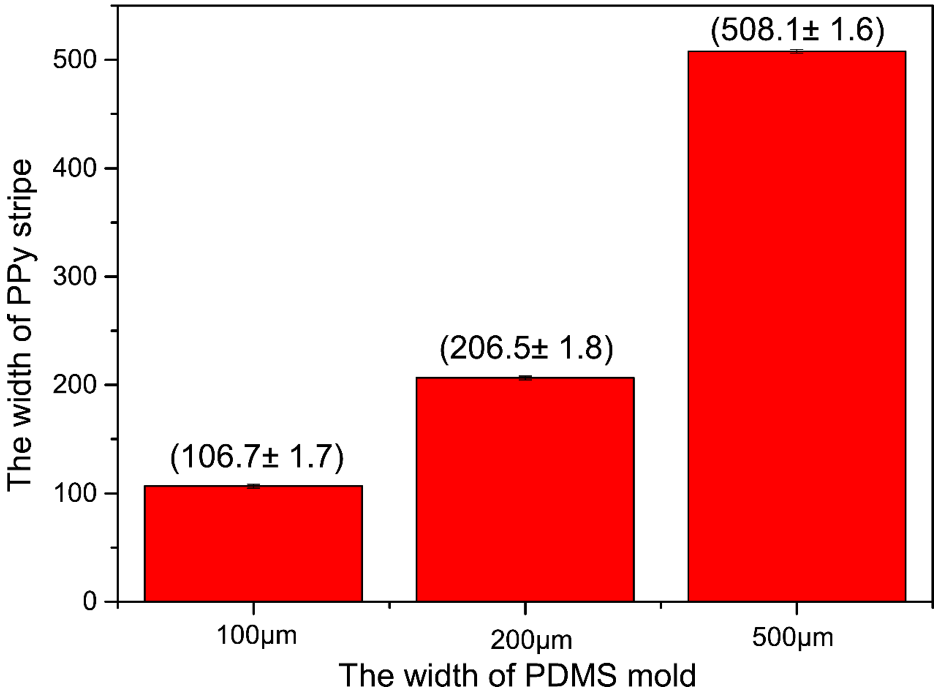

Supplementary Figure 2. The relationship between the width of PDMS mold and PPy stripe. The average width differences between PDMS mold (100, 200 and 500 μm in width) and corresponding PPy stripes (n=10) are 6.7 μm, 6.5 μm and 8.1 μm respectively. The width of PPy stripes are presented as mean (SD).

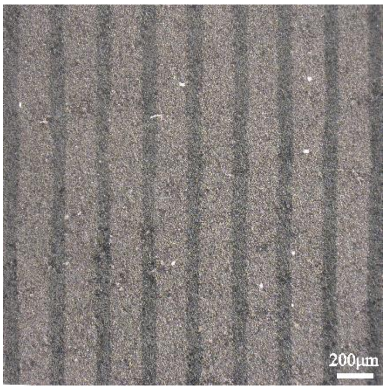

Supplementary Figure 3. The optical image of PPy stripes with 60 μm width.

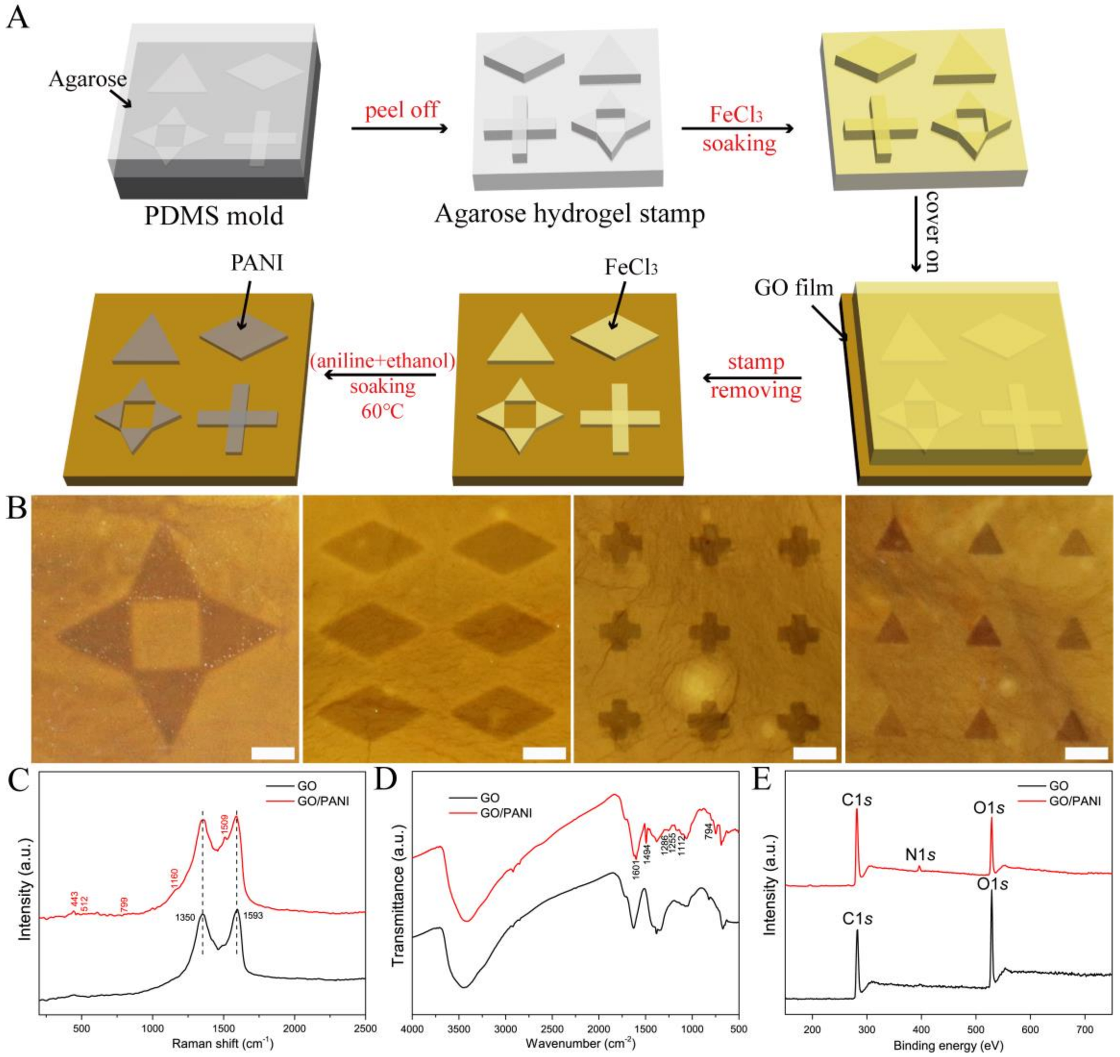

Supplementary Figure 4. The preparation and structural characterization of GO/PANI. (A) Schematic diagram of precise modification of PANI onto the GO film. (B) Optical images of PANI patterns with different sizes and shapes on the GO film; (C) Raman spectra of GO and GO/PANI; (D) FT-IR spectra of pure GO and a GO/PANI; (E) XPS spectra of GO and GO/PANI (Scale bar: 5 mm).

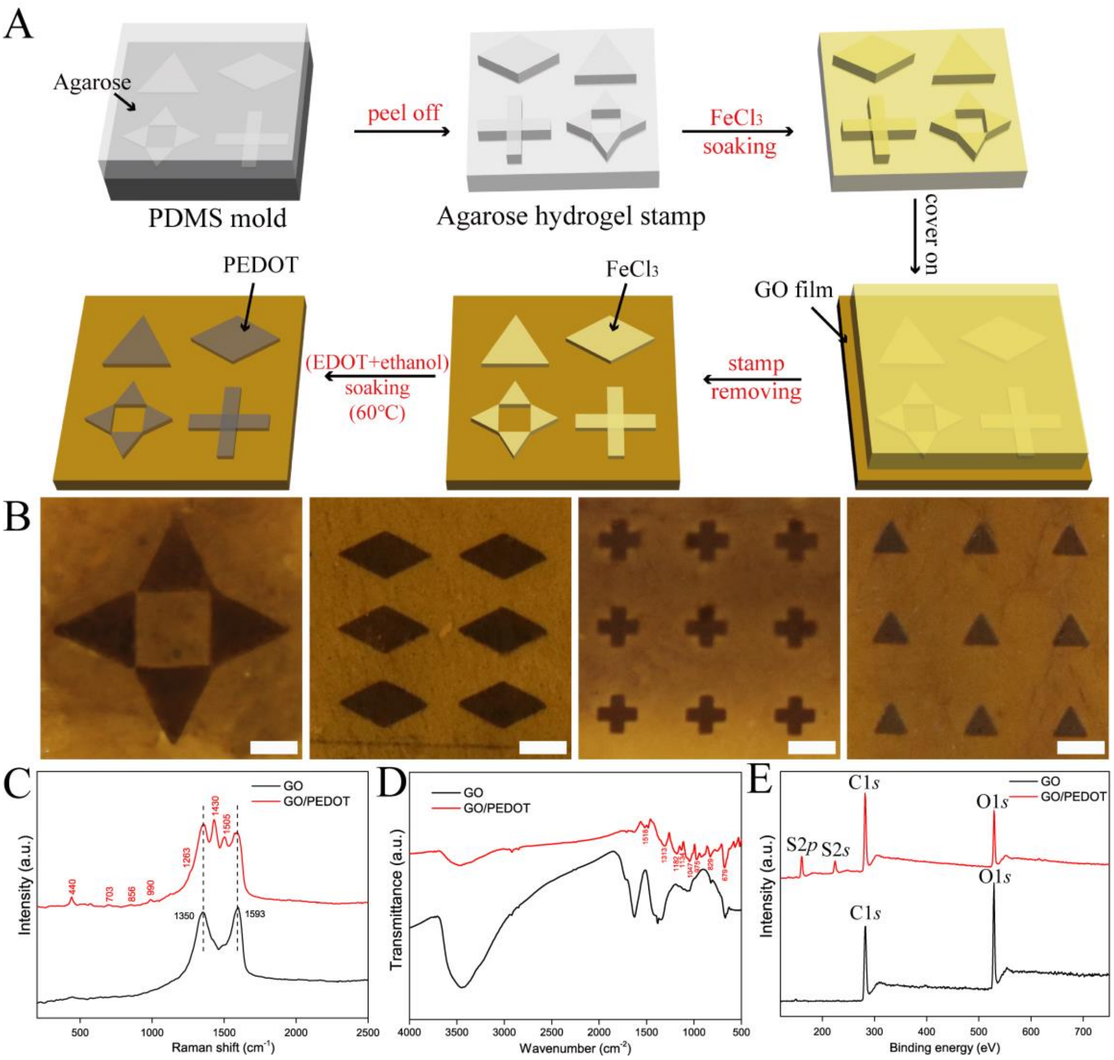

Supplementary Figure 5. The preparation and structural characterization of GO/PEDOT. (A) Schematic diagram of precise modification of PEDOT onto the GO film. (B) Optical images of PEDOT patterns with different sizes and shapes on the GO film; (C) Raman spectra of GO and GO/ PEDOT; (D) FT-IR spectra of pure GO and a GO/ PEDOT. (E) XPS spectra of GO and GO/ PEDOT (Scale bar: 5 mm).

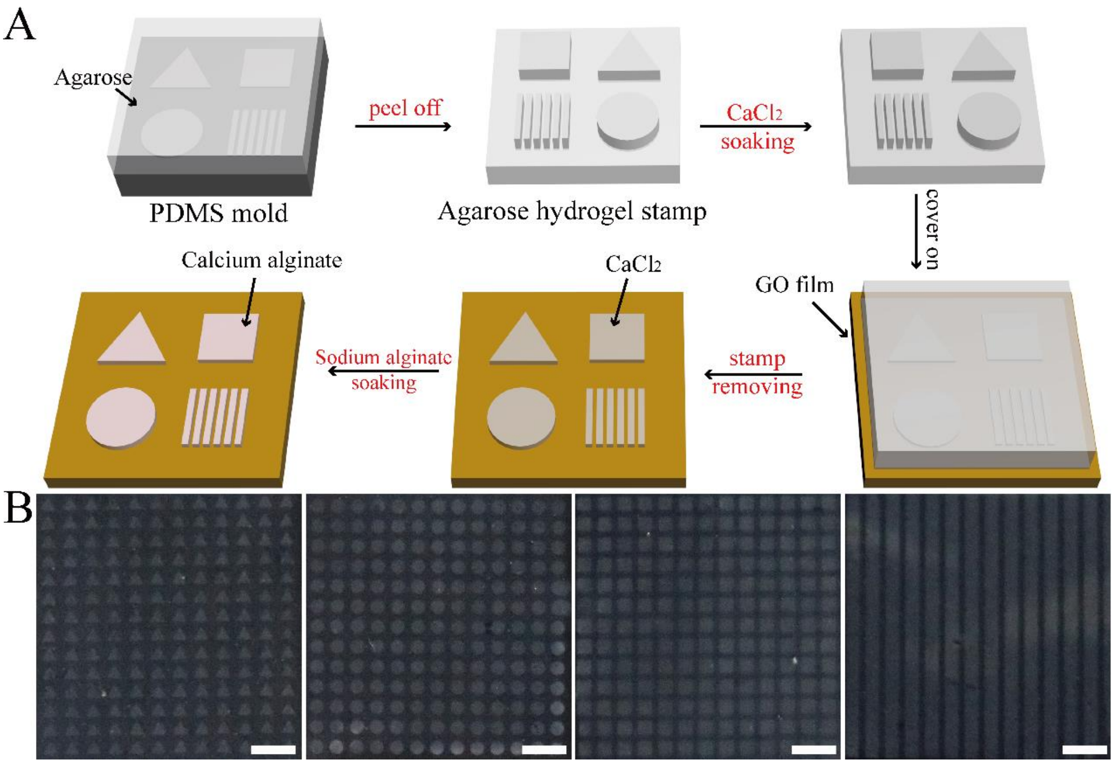

Supplementary Figure 6. The preparation and structural characterization of calcium alginate hydrogel/GO. (A) Schematic diagram of precise modification of calcium alginate hydrogel onto the GO film. (B) Optical images of calcium alginate hydrogel patterns with different sizes and shapes on the GO film (Scale bar: 2 mm).

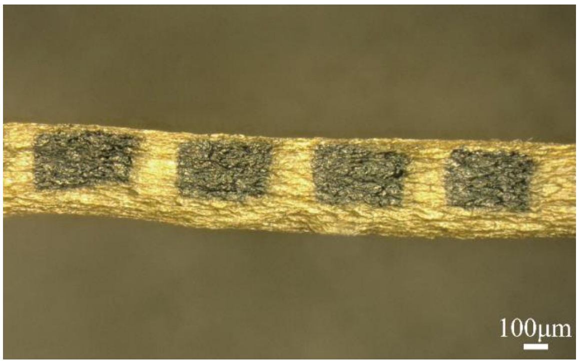

Supplementary Figure 7. PPy patterning on GO fiber, the precise modification of PPy on GO fiber can be achieved easily.

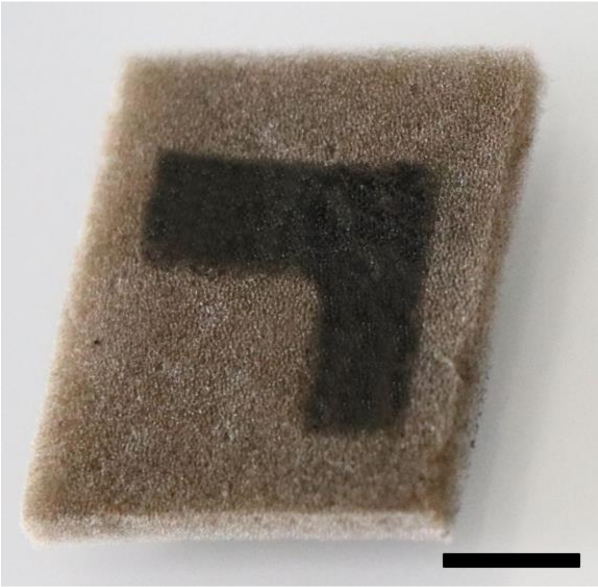

Supplementary Figure 8. PPy patterning on GO foam (Scale bar: 1 cm).

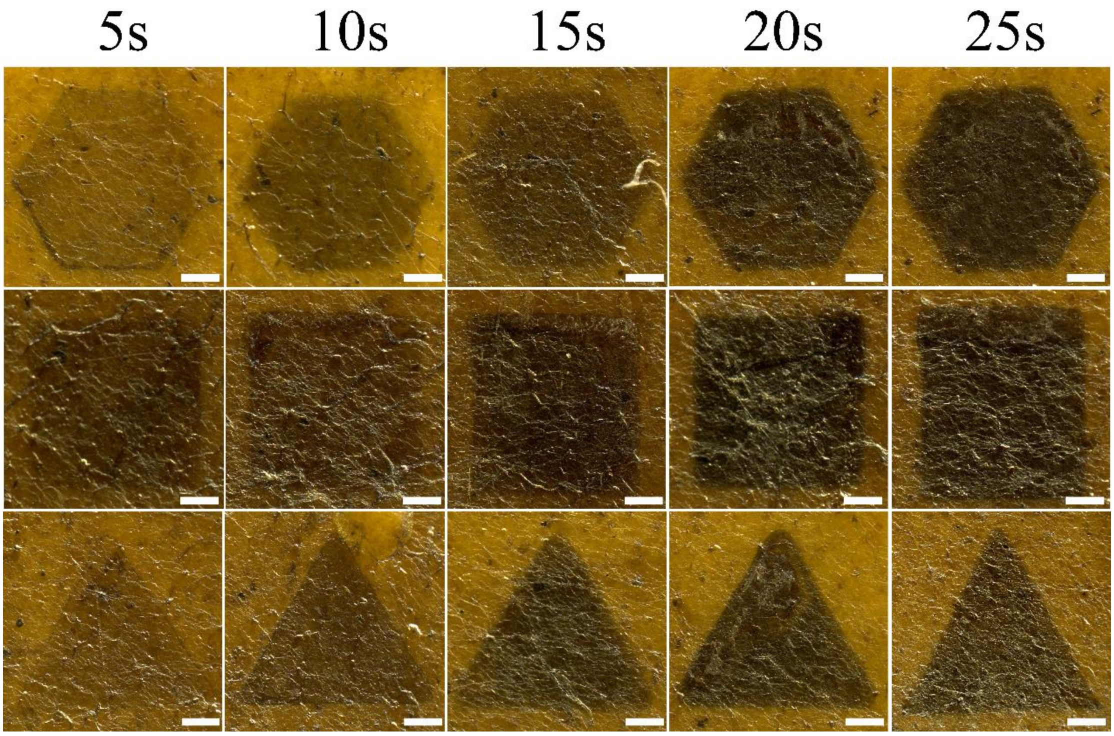

Supplementary Figure 9. PPy pattern with different microcontact time. The color of PPy patterns darkens gradually with the increasing microcontact time (Scale bar: 100 μm).

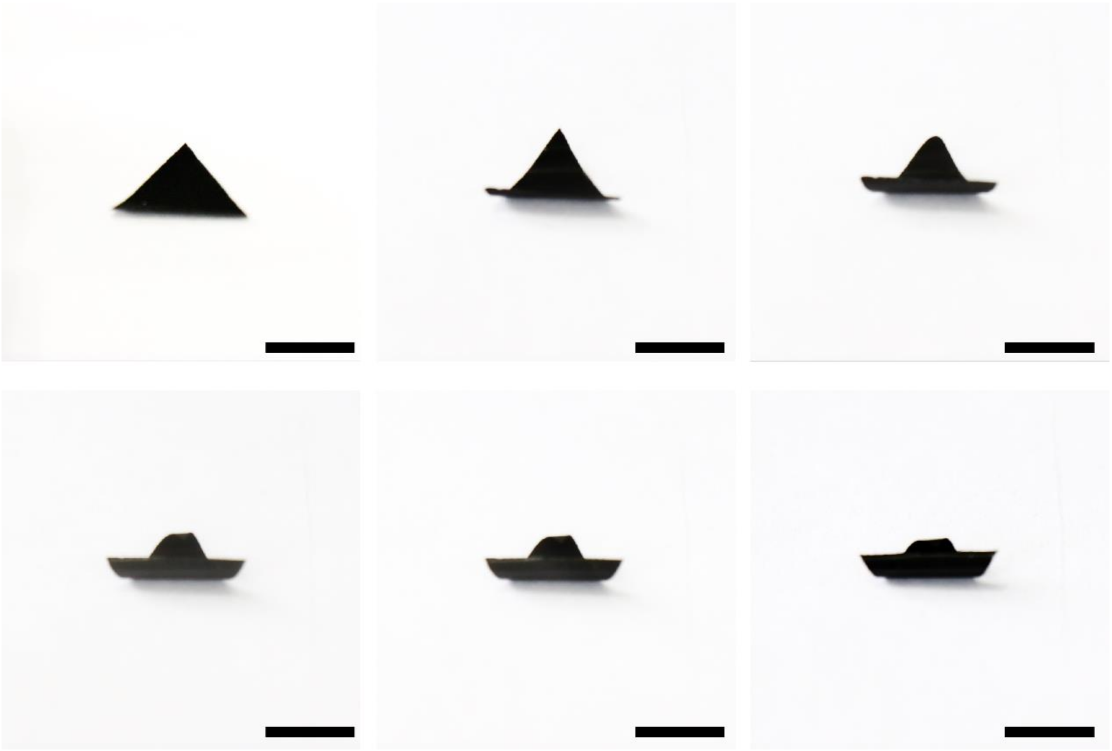

Supplementary Figure 10. The structure change process of trigonal GO/PPy actuator under the stimulus of humidity (Scale bar: 1 cm).

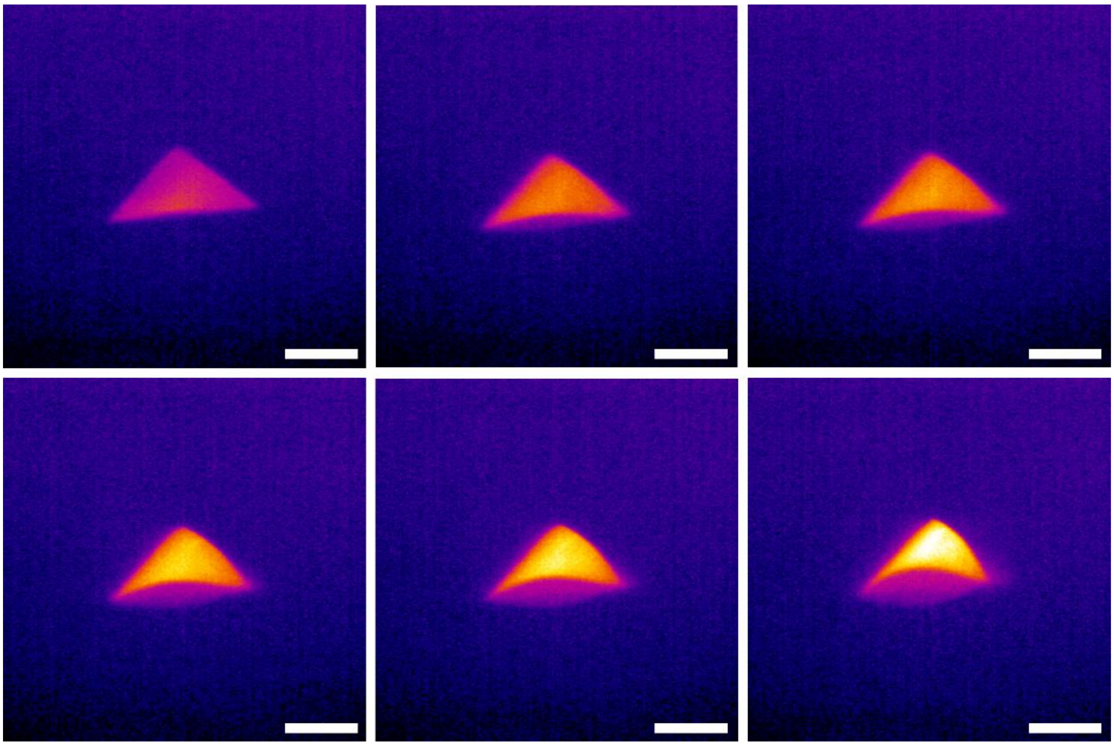

Supplementary Figure 11. The structure change process of trigonal GO/PPy actuator under the stimulus of IR light (Scale bar: 1 cm).

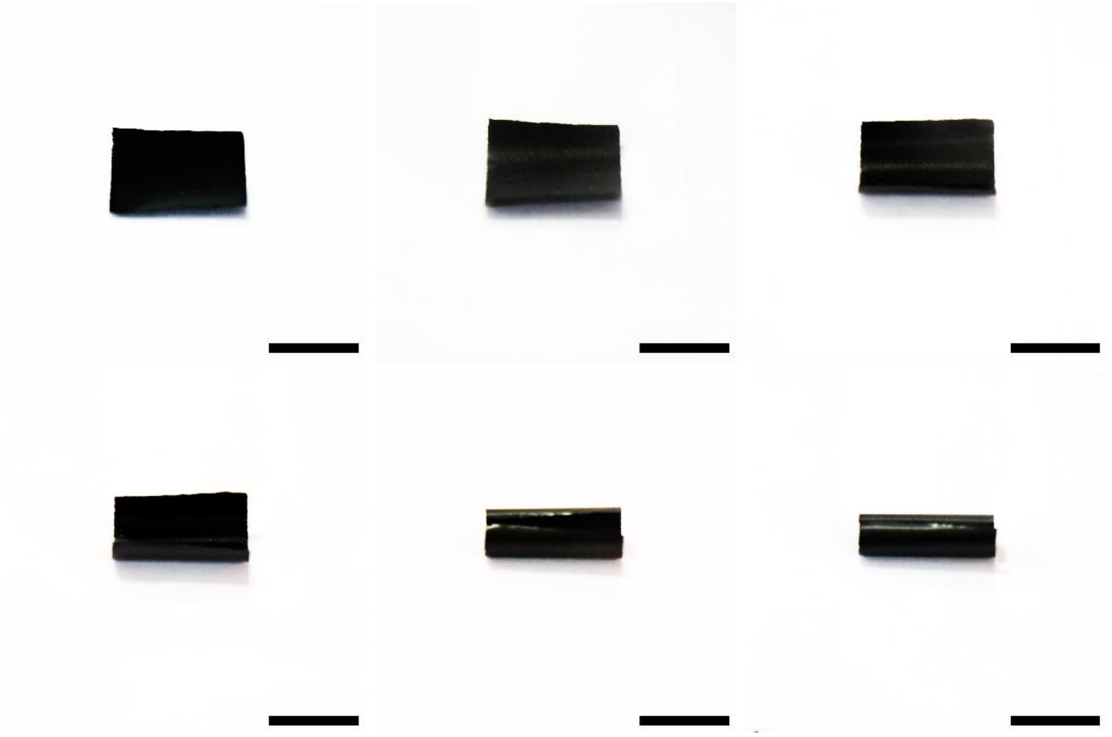

Supplementary Figure 12. The structure change process of rectangular GO/PPy actuator under the stimulus of humidity (Scale bar: 1 cm).

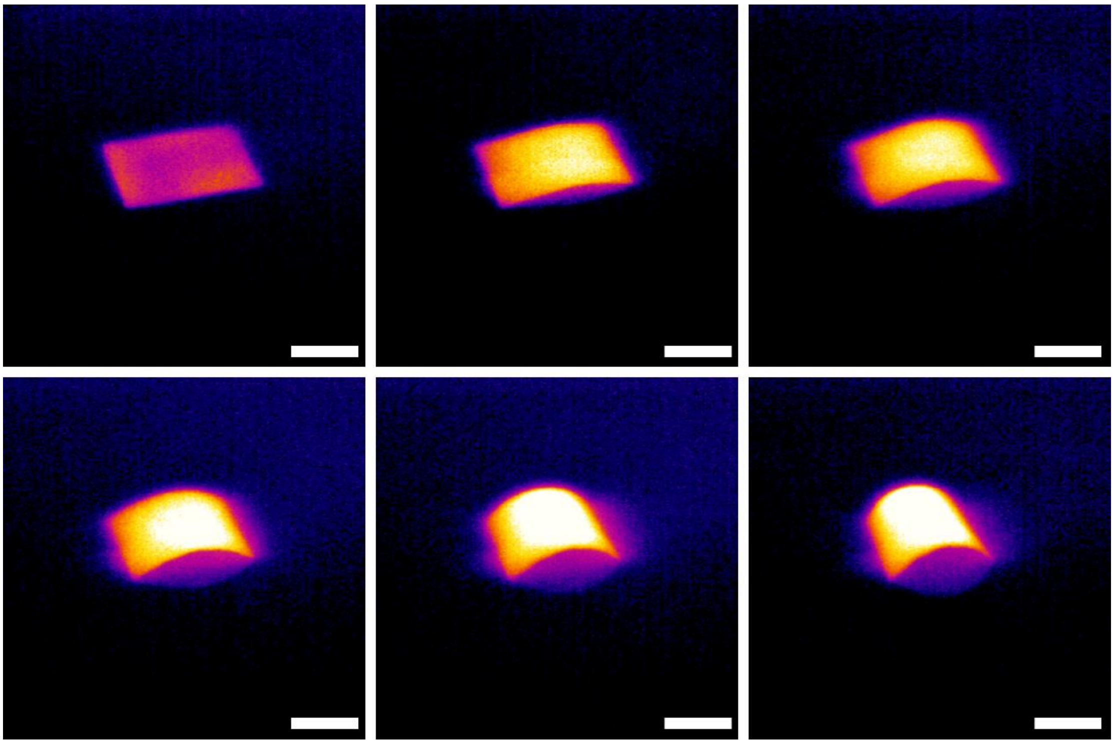

Supplementary Figure 13. The structure change process of rectangular GO/PPy actuator under the stimulus of IR light (Scale bar: 1 cm).

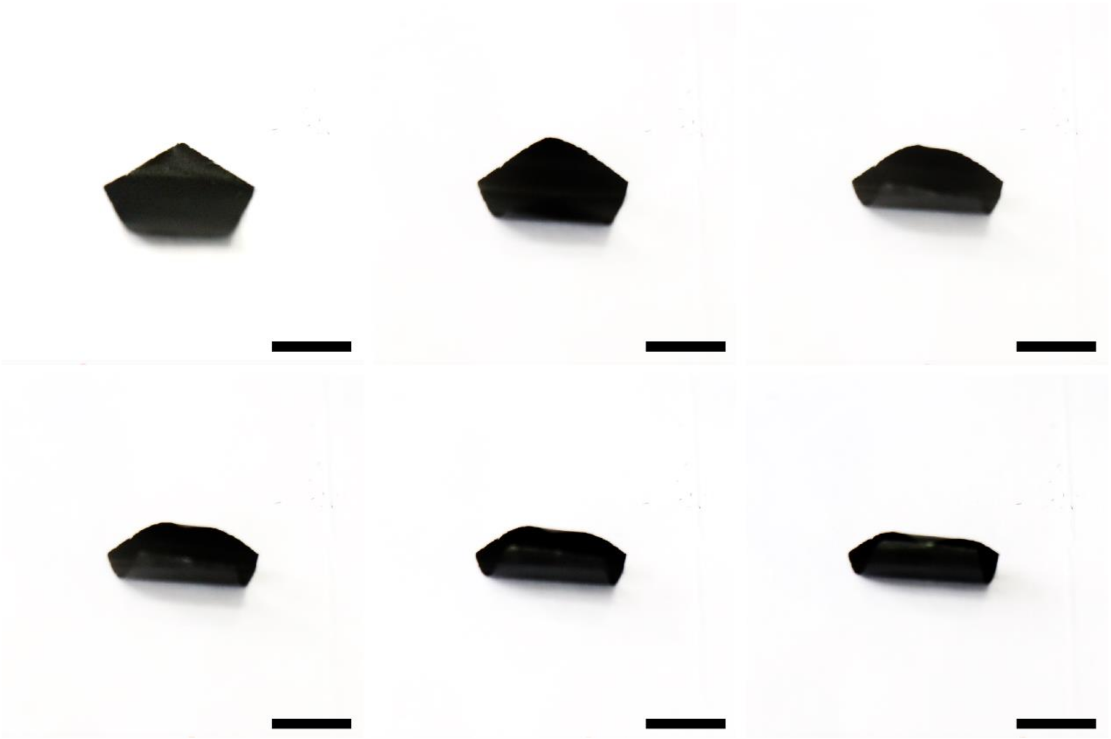

Supplementary Figure 14. The structure change process of pentagonal GO/PPy actuator under the stimulus of humidity (Scale bar: 1 cm).

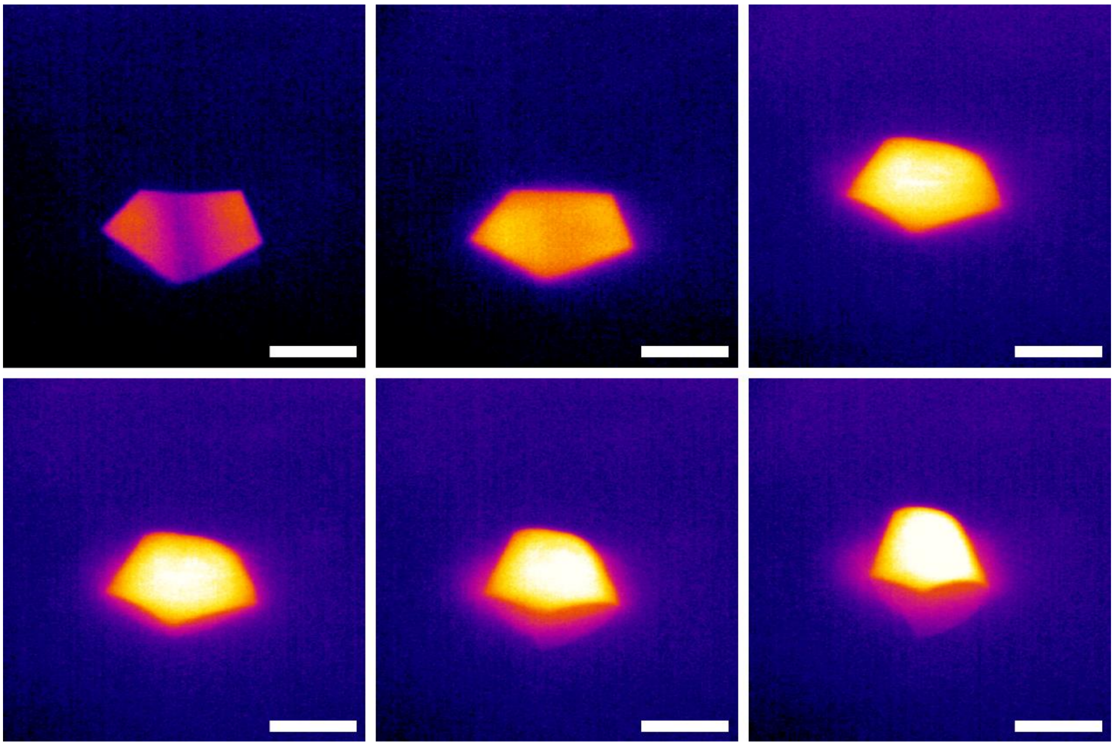

Supplementary Figure 15. The structure change process of pentagonal GO/PPy actuator under the stimulus of IR light (Scale bar: 1 cm).

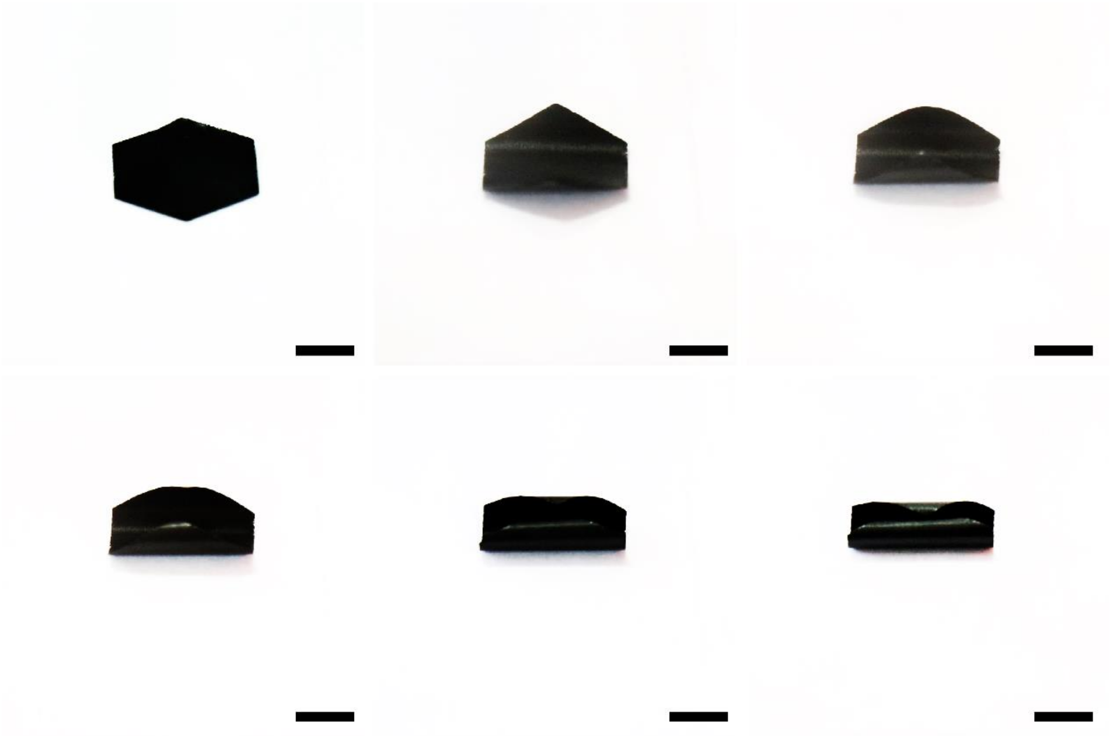

Supplementary Figure 16. The structure change process of hexagonal GO/PPy actuator under the stimulus of humidity (Scale bar: 1 cm).

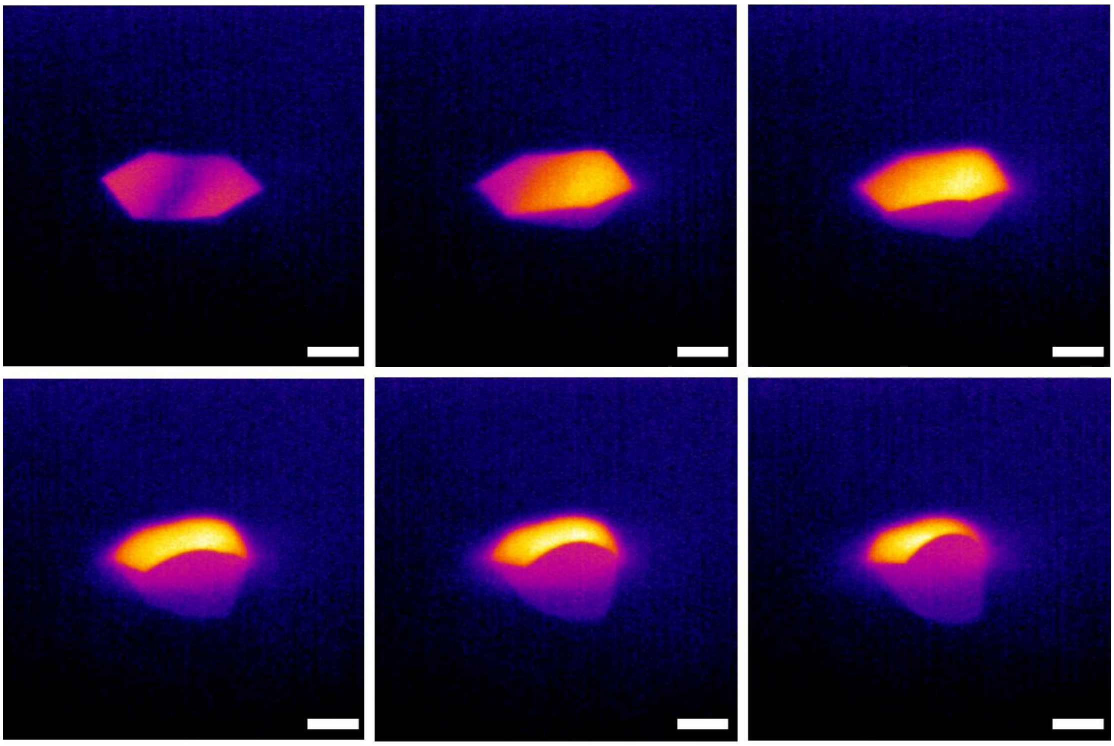

Supplementary Figure 17. The structure change process of hexagonal GO/PPy actuator under the stimulus of IR light (Scale bar: 1 cm).

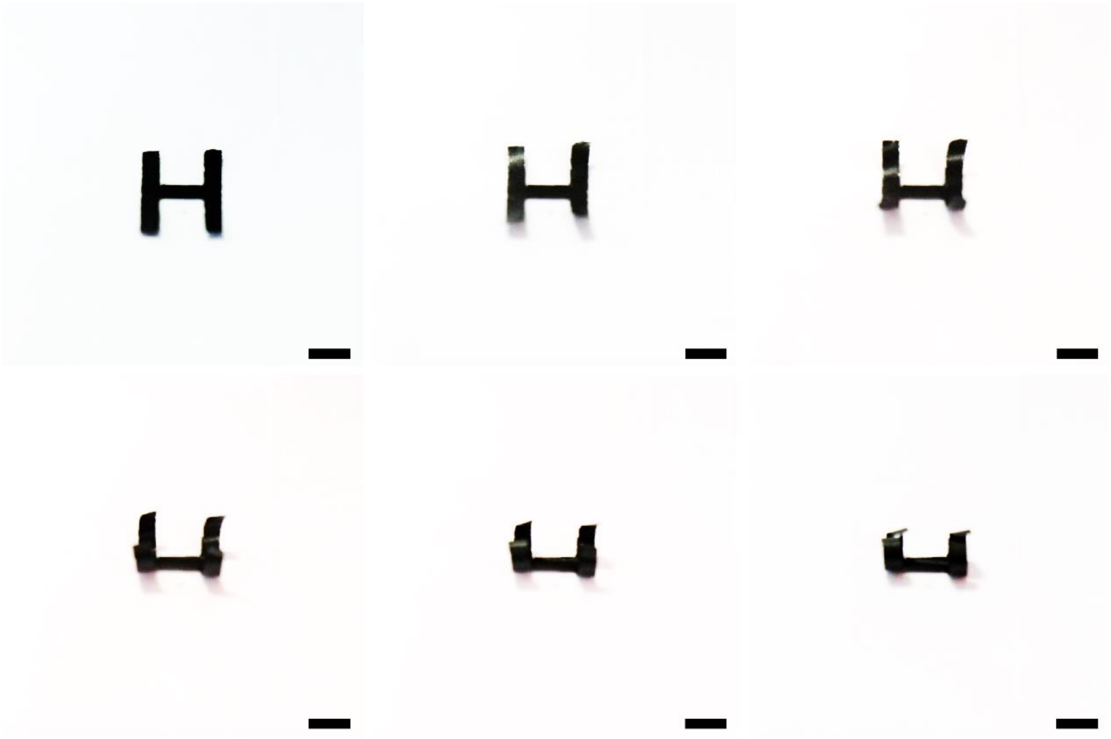

Supplementary Figure 18. The structure change process of H-like GO/PPy actuator under the stimulus of humidity (Scale bar: 1 cm).

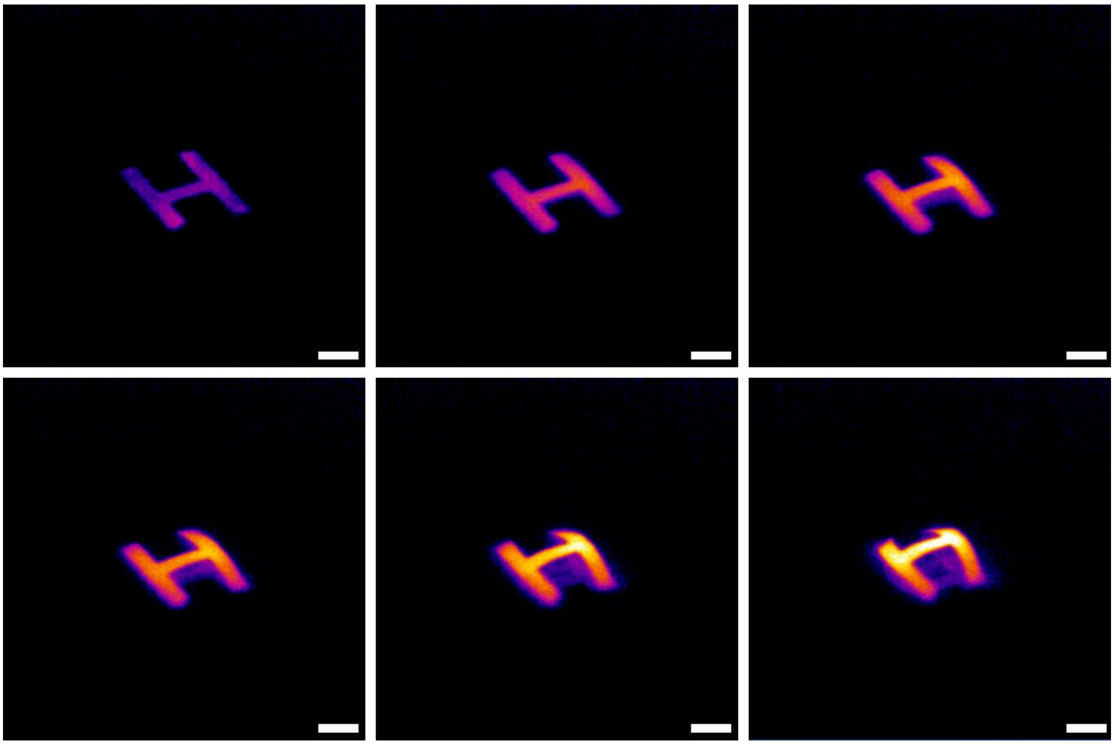

Supplementary Figure 19. The structure change process of H-like GO/PPy actuator under the stimulus of IR light (Scale bar: 1 cm).

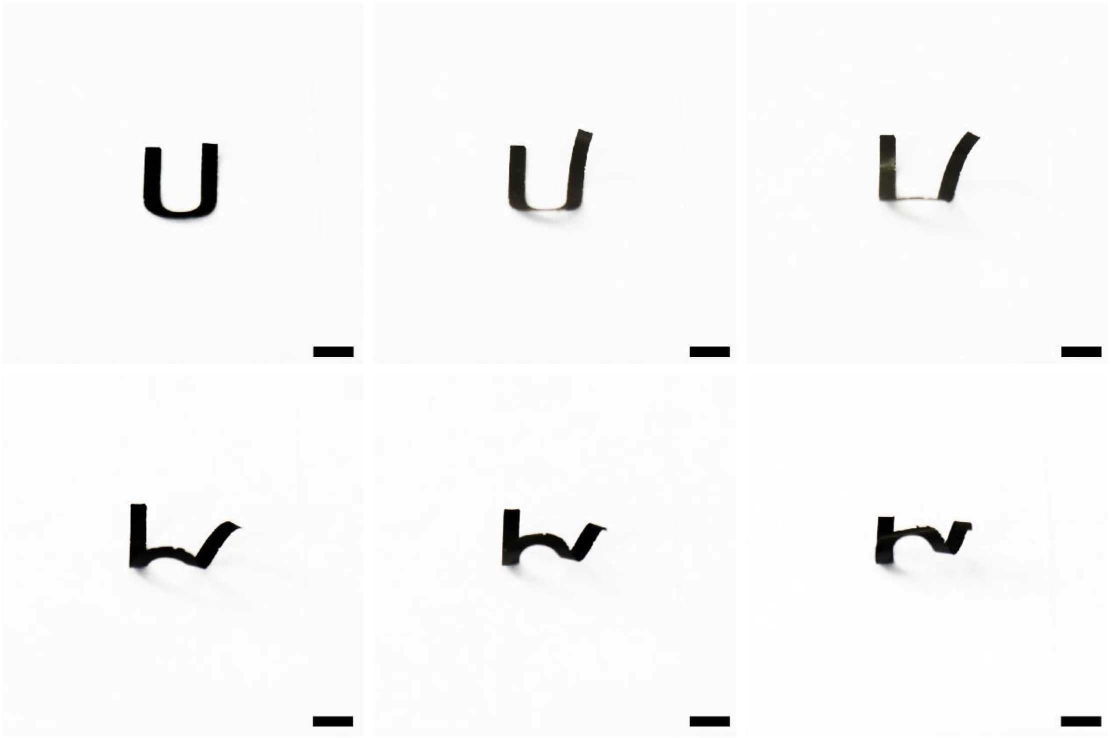

Supplementary Figure 20. The structure change process of U-like GO/PPy actuator under the stimulus of humidity (Scale bar: 1 cm).

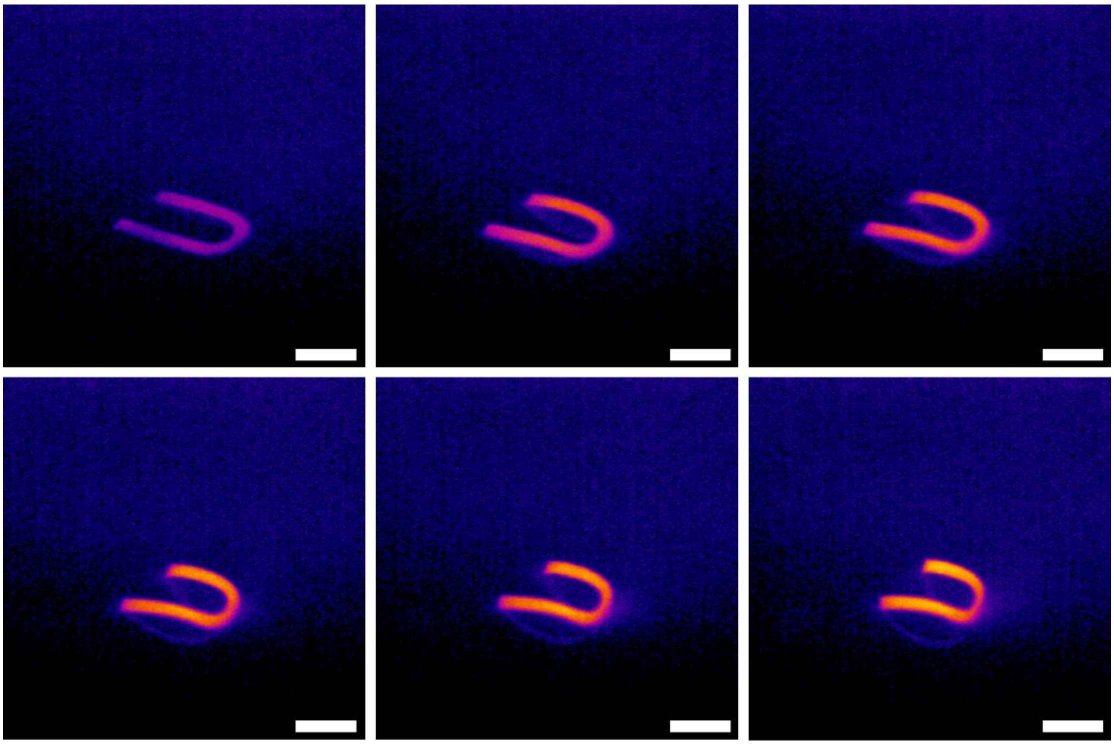

Supplementary Figure 21. The structure change process of U-like GO/PPy actuator under the stimulus of IR light (Scale bar: 1 cm).

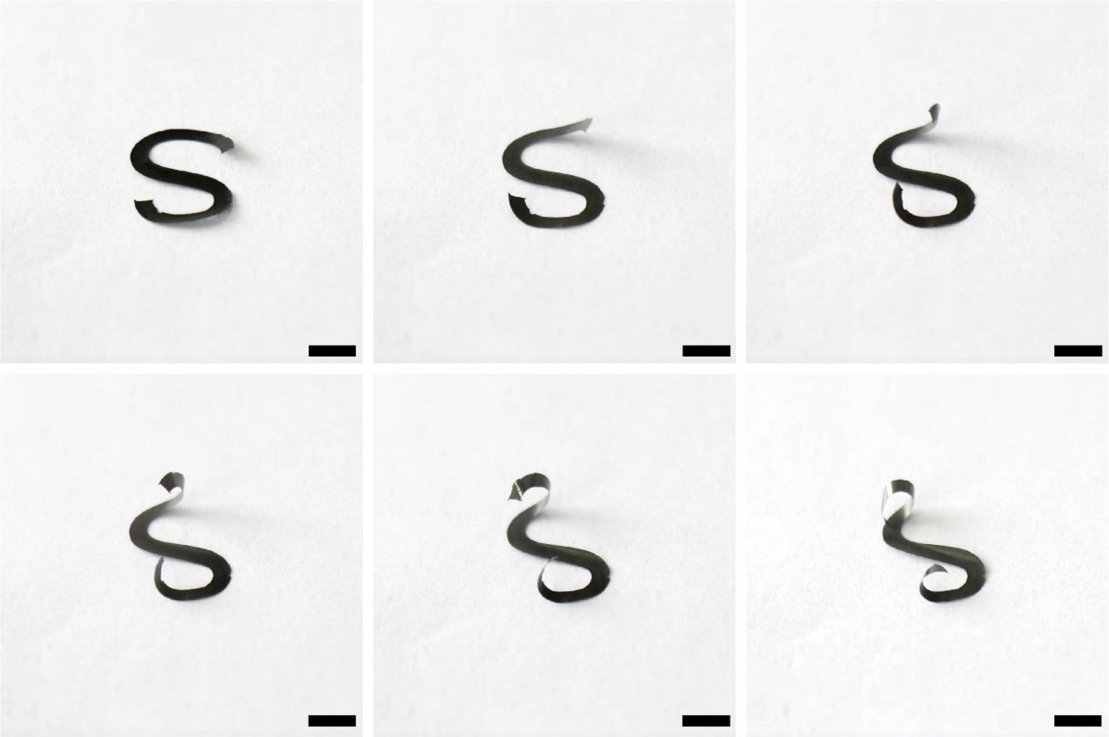

Supplementary Figure 22. The structure change process of S-like GO/PPy actuator under the stimulus of humidity (Scale bar: 1 cm).

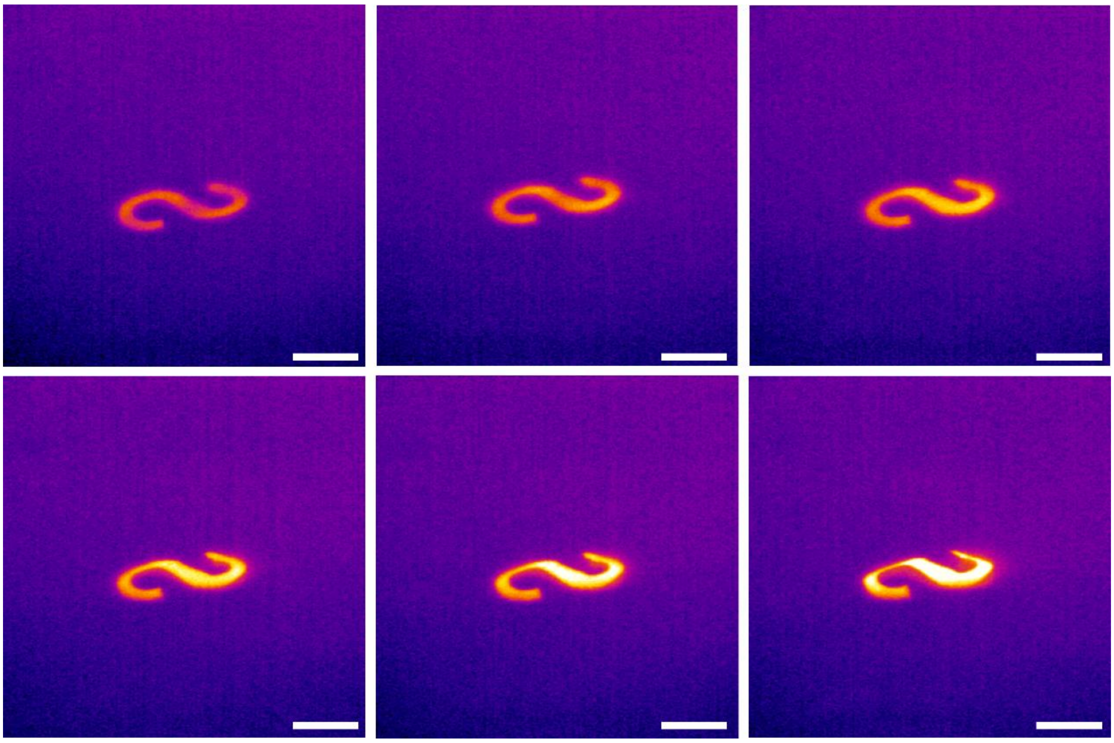

Supplementary Figure 23. The structure change process of S-like GO/PPy actuator under the stimulus of IR light (Scale bar: 1 cm).

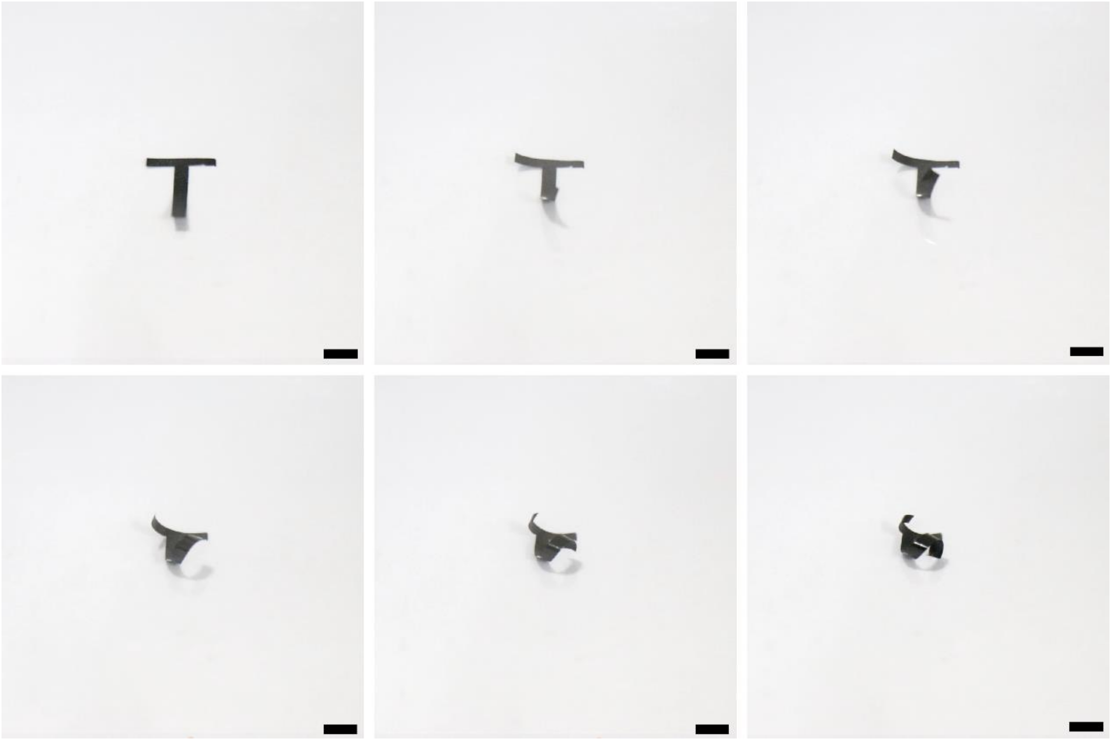

Supplementary Figure 24. The structure change process of T-like GO/PPy actuator under the stimulus of humidity (Scale bar: 1 cm).

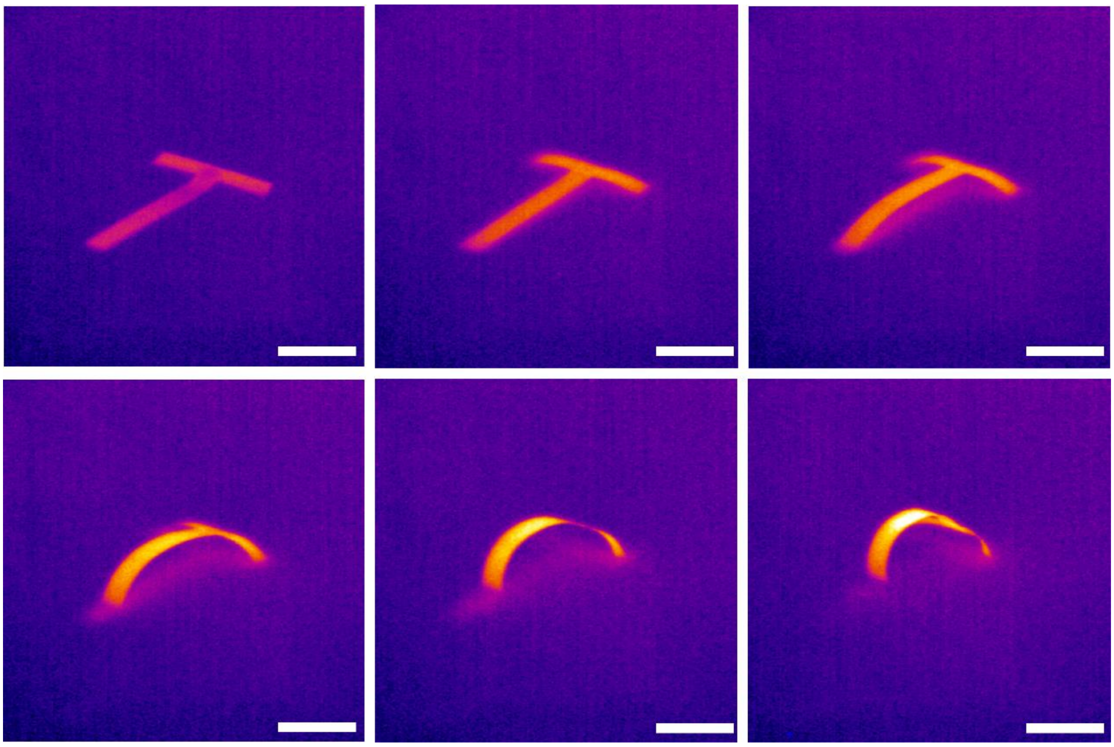

Supplementary Figure 25. The structure change process of T-like GO/PPy actuator under the stimulus of IR light (Scale bar: 1 cm).

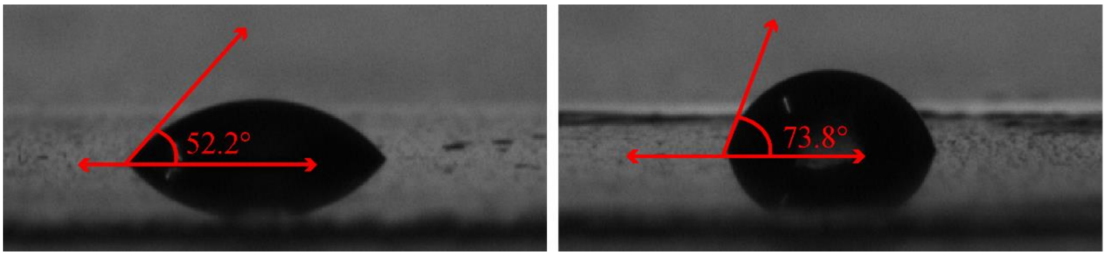

Supplementary Figure 26. Photos of water droplet contact angles for GO film (left) and GO/PPy film (right). The polymer of PPy can increase the hydrophobicity of GO film, so the water droplet contact angle for GO film is larger than GO/PPy film.

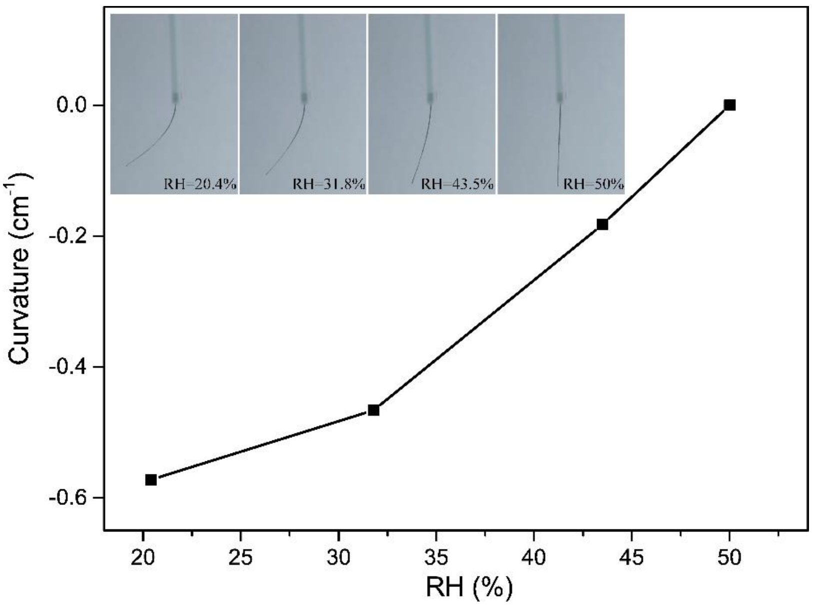

Supplementary Figure 27. Actuating performance of GO/PPy (b) at humidity levels from 20% to 50%. The insets in Figure S27 show photographs of the bending GO/PPy (b) at humidity levels of 20.4%, 31.8%, 43.5% and 50%.

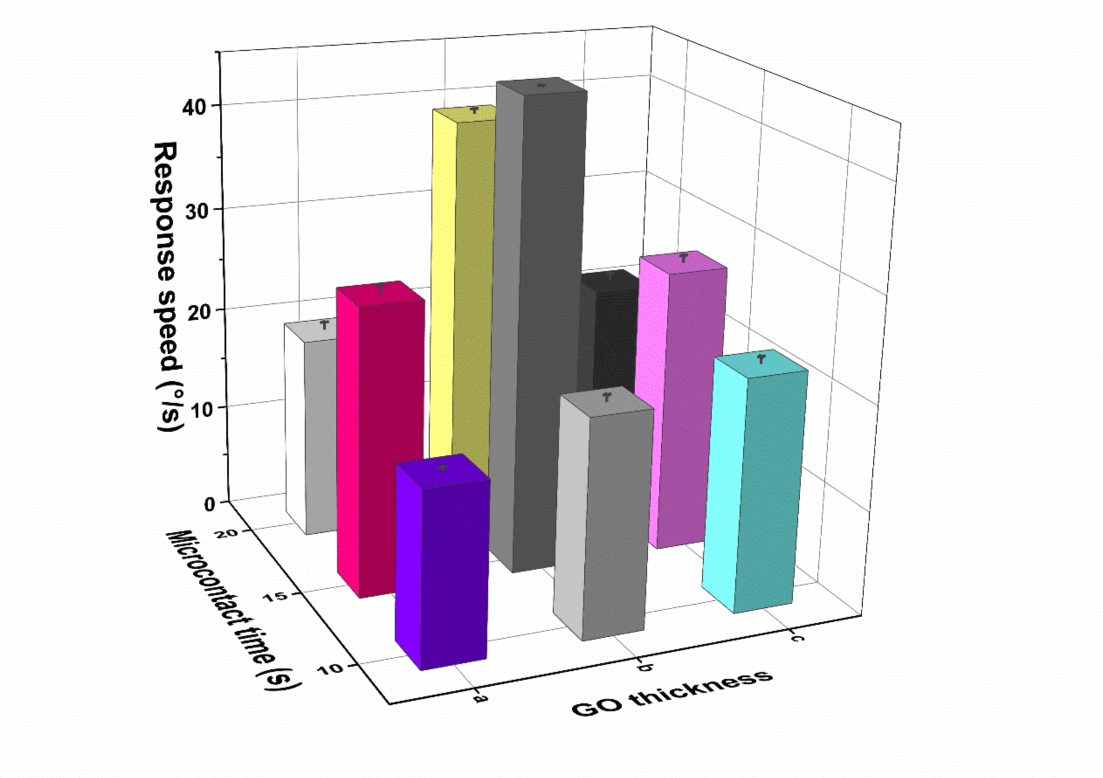

Supplementary Figure 28. The relationship between the response speed of the actuators (width 2 mm and length 15 mm) and the thicknesses of two layers, a, b, c represent the GO thicknesses of 12.8 μm, 25.2 μm and 37.1 μm respectively, and the PPy thickness increases gradually with the increasing microcontact time and the contact time of 10 s, 15 s and 20 s are named as GO/PPy (d), GO/PPy (e) and GO/PPy (b), respectively. Respond speed are presented as mean (SD).

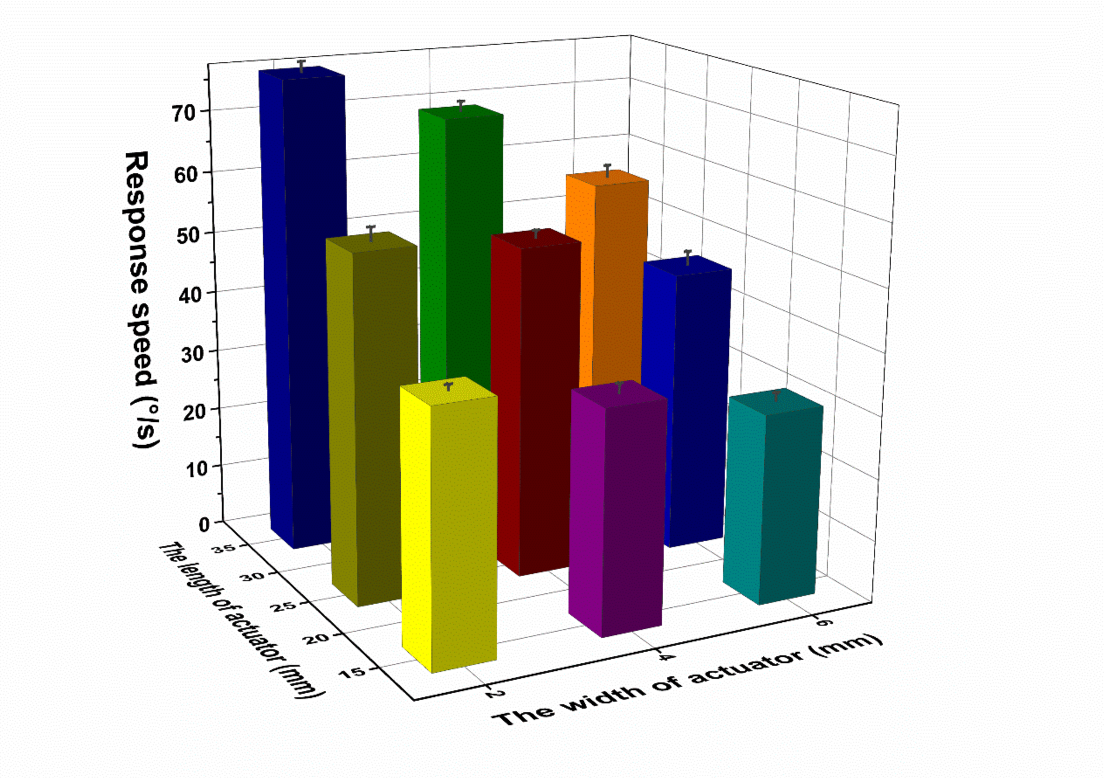

Supplementary Figure 29. The relationship between the response speed of the actuators (GO thickness of 25.2 μm and microcontact time of 20 s) and their sizes. We find clearly that the response speed increases gradually with the increasing actuator length at the same width, and the response speed decreases gradually with the increasing actuator width, which is ascribed to the larger bending force for the larger actuator length and larger flexural rigidity for the larger width of actuator [1]. Respond speed are presented as mean (SD).

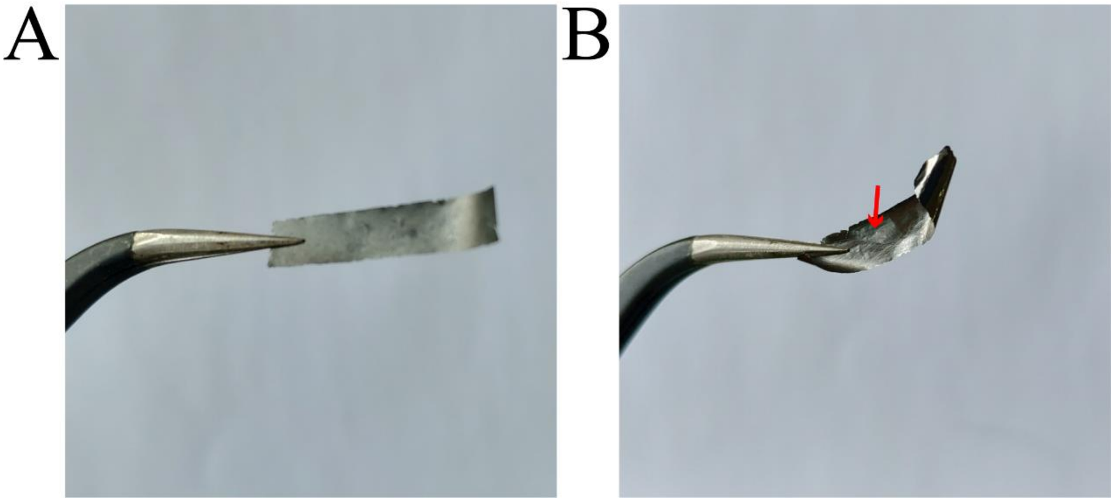

Supplementary Figure 30. The morphology change of GO/PPy with 120 °C treatment and without treatment. (A) GO/PPy without treatment, (B) with 120 °C treatment (the red arrow indicates the exfoliation of PPy from GO film).

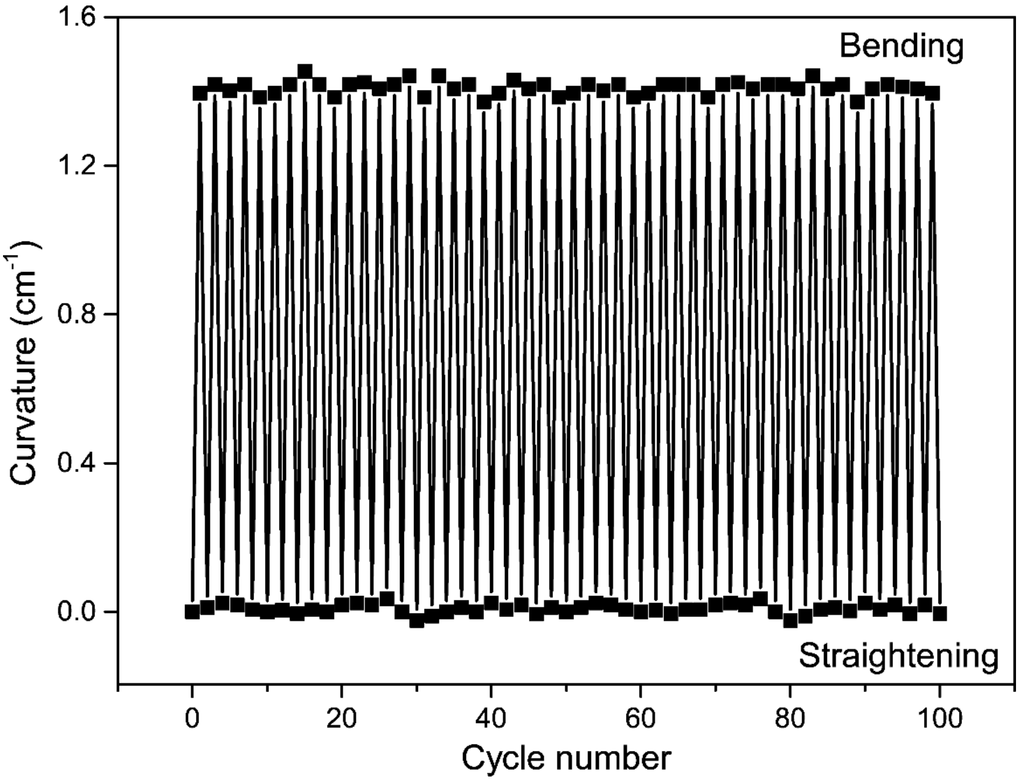

Supplementary Figure 31. Cycle stability of curvature for GO/PPy actuator over 100 cycles under the stimulus of IR. The GO/PPy actuator display excellent cycle stability.

Supplementary Note 1

Discussion for Supplementary Figure 4:

Supplementary Figure 4A showed the schematic diagram of precise modification of PANI onto the GO film. Similar to the modification of PPy, $FeCl_3$ was transferred onto the GO film by a $FeCl_3$-loaded agarose hydrogel stamp, and $FeCl_3$ was present in only specific patterns replicated from the agarose hydrogel stamp. After that, the mixture of aniline and ethanol (V (aniline): V (ethanol) = 3:2) was dropped onto the GO film and reacted with $FeCl_3$ to generate PANI with specific patterns on the GO film. Raman, FT-IR and XPS test were conducted to demonstrate the successful modification of PANI on the GO film. Supplementary Figure 4B exhibited the optical images of PANI patterns with different sizes and shapes on the GO film. We can see clearly that light black PANI patterns are introduced onto the GO film with high precision. Supplementary Figure 4C displayed the Raman spectra of GO and GO/PANI. The spectrum of GO has two dominant peaks at 1350 and 1593 cm^{-1}, corresponding to its *D* and *G* bands. Compared to the Raman spectrum of GO, some characteristic Raman bands of PANI appeared in the Raman spectrum of PANI. The Raman band at 443 cm^{-1} is associated with the phenazine-like segment. The band related to out-of-plane C-H deformation of quinonoid ring appears at around 512 cm^{-1}. The 799 and 1160 cm^{-1} bands are assigned to the C-H bending in quinonoid ring and C–H bending deformation in the benzenoid ring. The 1509 cm^{-1} band is associated with C=N stretching vibrations of the quinonoid units. The appearance of the above bands suggests the formation of PANI on the surface of GO film [2-3]. In order to further confirm the formation of PANI on the surface of GO film, the FT-IR analysis was also employed to characterize the GO/PANI. Supplementary Figure 4D, a group of typical bands of PANI appeared [2, 4], C=N stretching of the quinonoid ring and C=C stretching of the benzenoid ring at 1601 and 1494 cm^{-1} respectively, C–N stretching of secondary aromatic amines at 1286 cm^{-1}, C–H bendings of the benzenoid ring and the quinonoid ring at 1245 and 1124 cm^{-1} respectively, and C-C stretching of the quinonoid at 794 cm^{-1}. The XPS spectra revealed the surface element compositions of GO and GO/PANI, exhibiting bands at 281.2, 396.4 and 529.8 eV, corresponding to C1*s*, N1*s*, and O1*s*, respectively (Supplementary Figure 4E). The existence of nitrogen element for GO/PANI also demonstrate the formation of PANI on the GO film.

Supplementary Note 2

Discussion for Supplementary Figure 5:

Supplementary Figure 5A showed the schematic diagram of precise modification of PEDOT onto the GO film. Similar to the modification of PPy and PANI, $FeCl_3$ was transferred onto the GO film by a $FeCl_3$-loaded agarose hydrogel stamp, and $FeCl_3$ was present in only specific patterns replicated from the agarose hydrogel stamp. After that, the mixture of EDOT and ethanol (V (EDOT): V (ethanol) = 4:1) was dropped onto the GO film and reacted with $FeCl_3$ to generate PEDOT with specific patterns on the GO film. Raman, FT-IR and XPS test were conducted to demonstrate the successful modification of PEDOT on the GO film. Supplementary Figure 5B exhibited the optical images of PEDOT patterns with different sizes and shapes on the GO film. We can see clearly that black PEDOT patterns are introduced onto the GO film with high precision. Supplementary Figure 5C displayed the Raman spectra of GO and GO/PEDOT. The spectrum of GO has two dominant peaks at 1350 and 1593 cm^{-1}, corresponding to its *D* and *G* bands. Compared to the Raman spectrum of GO, some characteristic Raman bands of PEDOT appeared in the Raman spectrum of PEDOT. The Raman bands at 1430 and 1505 cm^{-1} are attributed to the symmetric and asymmetric stretching vibration of the C=C bond in PEDOT, respectively. The band at 1263cm^{-1} is assigned to the stretching modes of single C-C inter-ring bonds in PEDOT. Other weaker bands at around 990, 856 and 703 cm^{-1} are assigned to C-C asymmetric bond, C–H bending of 2, 3, 5-trisubstituted thiophene and C-S-C bond of PEDOT. The appearance of the above bands suggests the formation of PEDOT on the surface of GO film [5-7]. In order to further confirm the formation of PEDOT on the surface of GO film, the FT-IR analysis was also employed to characterize the GO/PEDOT. As shown in Supplementary Figure 5D, a group of typical bands of PEDOT appeared [8-9], the feature peaks of PEDOT at about 1518 and 1313 cm^{-1} (C=C and C-C stretching vibrations of the quininoid structure of the thiophene ring) demonstrate the presence of PEDOT. The other bands at about 1182, 1134, and 1047 cm^{-1} are attributed to the C-O-C bond stretching, and the C–S bond in thethiophene ring is proved by the presence of bands at about 975, 829 and 679 cm^{-1}. The XPS spectra revealed the surface element compositions of GO and GO/PEDOT, exhibiting bands at 161.3, 225.6, 281.2 and 529.8 eV, corresponding to S2*p*, S2*s*, C1*s* and O1*s*, respectively (Supplementary Figure 5E). The existence of surfur element for GO/PEDOT also demonstrate the formation of PEDOT on the GO film.

Supplementary Note 3

Discussion for Supplementary Figure 6:

Supplementary Figure 6A showed the schematic diagram of precise modification of calcium alginate onto the GO film. Similar to the modification of PPy, PANI and PEDOT, $CaCl_2$ was transferred onto the GO film by a $CaCl_2$-loaded agarose hydrogel stamp, and $CaCl_2$ was present in only specific patterns replicated from the agarose hydrogel stamp. After that, 2% sodium alginate was dropped onto the GO film and reacted with $CaCl_2$ to generate calcium alginate with specific patterns on the GO film.

Supplementary Figure 6B exhibited the optical images of calcium alginate patterns with different sizes and shapes on the GO film. We can see clearly that grey white calcium alginate patterns are introduced onto the GO film with high precision.

Supplementary Note 4

Discussion for Supplementary Figure 28:

Supplementary Figure 28 exhibits The relationship between the response speed of the actuators (width 2 mm and length 15 mm) and the thicknesses of GO and PPy layers. We find that the actuators with the GO thickness of 25.2 μm possess larger response speed at the same microcontact time, this can be explained by the small water-adsorption-induced expansion force for actuator with the GO thickness of 12.8 μm can't bend the actuator quickly, and the large stiffness of actuator with the GO thickness of 37.1 μm cannot bend the actuator flexibly. Furthermore, the GO/PPy (e) possesses maximum response speed, which is slightly larger than GO/PPy (b). This is because the PPy thickness reduces with the decreasing microcontact time, so the stiffness of actuator reduces synchronously. However, GO/PPy (d) is smaller in response speed than GO/PPy (b), which is owing to the minor difference of water-adsorption ability between GO and PPy layer at the microcontact time of 10s. Therefore, the proper choice of GO thickness and microcontact time is important for the actuator.

Supplementary Note 5

Calculation of energy conversion efficiency:

The energy conversion efficiency (η) of a bilayer actuator can be defined as the total elastic energy generated by the GO/PPy (b) actuator divided by the input laser energy (Q_{Laser}). The elastic energy of our actuators can be calculated as follow [10-11]:

$$Q_{\text{Elastic}}=\frac{[E_2 t_2^{\,2}(3t_1+t_2)+E_1 t_1^{\,2}(3t_2+t_1)][E_1^{\,2}t_1^{\,4}+E_2^{\,2}t_2^{\,4}+2E_1E_2t_1t_2(2t_1^{\,2}+2t_2^{\,4}+3t_1t_2)]}{36E_1E_2t_1^{\,2}t_2^{\,2}(t_1+t_2)}\times\left(\frac{1}{r}\right)^2 V_{actuator} \quad (1)$$

where Q_{Elastic} is the total elastic energy generated by GO/PPy (b) actuator under the stimuli of IR light with power densities (ρ_{Laser}) of 0.0831 W cm^{-2}. $1/r$ is the curvature (1.40 cm^{-1}), r is the radius of curvature. t_1 and t_2 are the thickness of PPy layer (15.2 μm) and GO layer (37.2 μm) respectively. E_1 and E_2 are the Young's modulus of PPy layer (80 MPa) and GO layer (3.07 GPa) respectively. The Young's modulus of the GO layer is measured by an All-Electrodynamic Dynamic Test Instrument (Instron Model E1000, England). The Young's modulus of the PPy layer has been reported in many paper with the same value [12-13].

The calculated total elastic energy of GO/PPy (b) actuator is 0.00137 J.

The actuating time (ʊ) is 3s. A_{actuator} represent the area exposed on the IR light. The input laser energy applied to the actuator can be expressed as:

$$Q_{\text{Laser}}=\rho_{\text{Laser}}\times ʊ \times A_{\text{actuator}} \qquad (2)$$

The calculated input laser energy of GO/PPy (b) actuator is 0.07479 J.

Hence, the energy conversion efficiency η is given by:

$$\eta=\frac{Q_{\text{Elastic}}}{Q_{\text{Laser}}} \qquad (3)$$

The energy conversion efficiency η is then calculated to be 1.832 %.

Supplementary References:

(1) Alici, G.; Mui, B.; Cook, C. Bending modeling and its experimental verification for conducting polymer actuators dedicated to manipulation applications. *Sensors and Actuators A: Physical.* **126**, 396-404 (2006).

(2) Cong, H.-P.; Ren, X.-C.; Wang, P.; Yu, S.-H. Flexible graphene-polyaniline composite paper for high-performance supercapacitor. *Energy & Environmental Science.* **6**, 1185-1191 (2013).

(3) Zhou, Q.; Li, Y.; Huang, L.; Li, C.; Shi, G. Three-dimensional porous graphene/polyaniline composites for high-rate electrochemical capacitors. *Journal of Materials Chemistry A.* **2**, 17489-17494 (2014).

(4) Mishra, A. K.; Ramaprabhu, S. Functionalized graphene-based nanocomposites for supercapacitor application. *The Journal of Physical Chemistry C.* **115**, 14006-14013 (2011).

(5) Wang, W.; Lei, W.; Yao, T.; Xia, X.; Huang, W.; Hao, Q.; Wang, X. One-pot synthesis of graphene/SnO_2/PEDOT ternary electrode material for supercapacitors. *Electrochimica Acta.* **108**, 118-126 (2013).

(6) Garreau, S.; Louarn, G.; Buisson, J.; Froyer, G.; Lefrant, S. In situ spectroelectrochemical Raman studies of poly (3, 4-ethylenedioxythiophene)(PEDT). *Macromolecules.* **32**, 6807-6812 (1999).

(7) Selvaganesh, S. V.; Mathiyarasu, J.; Phani, K.; Yegnaraman, V. Chemical synthesis of PEDOTâ€“Au nanocomposite. *Nanoscale research letters.* **2**, 546 (2007).

(8) Sun, D.; Jin, L.; Chen, Y.; Zhang, J. R.; Zhu, J. J. Microwave-Assisted In Situ Synthesis of Graphene/PEDOT Hybrid and Its Application in Supercapacitors. *ChemPlusChem* **78**, 227-234 (2013).

(9) Zhou, H.; Yao, W.; Li, G.; Wang, J.; Lu, Y. Graphene/poly (3, 4-ethylenedioxythiophene) hydrogel with excellent mechanical performance and high conductivity. *Carbon.* **59**, 495-502 (2013).

(10) Amjadi, M.; Sitti, M. High-performance multiresponsive paper actuators. *ACS nano.* **10**, 10202-10210 (2016).

(11) Chen, L.; Weng, M.; Zhang, W.; Zhou, Z.; Zhou, Y.; Xia, D.; Li, J.; Huang, Z.; Liu, C.; Fan, S. Transparent actuators and robots based on single-layer superaligned carbon nanotube sheet and polymer composites. *Nanoscale.* **8**, 6877-6883 (2016).

(12) Fang, Y.; Tan, X.; Shen, Y.; Xi, N.; Alici, G. A scalable model for trilayer conjugated polymer actuators and its experimental validation. *Materials Science and Engineering: C.* **28**, 421-428 (2008).

(13) Alici, G. r.; Metz, P.; Spinks, G. M. A methodology towards geometry optimization of high performance polypyrrole (PPy) actuators. *Smart materials and structures.* **15**, 243 (2006).
