# Supplementary material for: Multi-stimuli-responsive programmable biomimetic actuator
Source: Nat Commun. 2019 Sep 9;10:4087. doi: 10.1038/s41467-019-12044-5 (PMC6733902; doi:10.1038/s41467-019-12044-5)
Supplement: Supplementary file 3 — Description of Additional Supplementary Files [file 41467_2019_12044_MOESM3_ESM.pdf]

Description of Additional Supplementary Files

File Name: Supplementary Movie 1
Description: Actuating performance of GO/PPy(a) under the stimulus of humidity; Actuating performance of GO/PPy(b) under the stimulus of humidity; Actuating performance of GO/PPy(c) under the stimulus of humidity

File Name: Supplementary Movie 2
Description: Actuating performance of GO/PPy(b) under the stimulus of IR light

File Name: Supplementary Movie 3
Description: The process of foam's picking and release by smart GO/PPy gripper with cross structure under the stimulus of humidity.

File Name: Supplementary Movie 4
Description: The process of flower's picking from vase by smart GO/PPy gripper with helical structure under the stimulus of humidity

File Name: Supplementary Movie 5
Description: The movement of a soft walking robot under the interval stimulus of IR light.
